# Patterns of relapse in patients with localized gastric adenocarcinoma who had surgery with or without adjunctive therapy: costs and effectiveness of surveillance

**DOI:** 10.18632/oncotarget.19226

**Published:** 2017-07-13

**Authors:** Elena Elimova, Rebecca S. Slack, Hsiang-Chun Chen, Venkatram Planjery, Hironori Shiozaki, Yusuke Shimodaira, Nick Charalampakis, Quan Lin, Kazuto Harada, Roopma Wadhwa, Jeannelyn S. Estrella, Dilsa Mizrak Kaya, Tara Sagebiel, Jeffrey H. Lee, Brian Weston, Manoop Bhutani, Mariela Blum Murphy, Aurelio Matamoros, Bruce Minsky, Prajnan Das, Paul F. Mansfield, Brian D. Badgwell, Jaffer A. Ajani

**Affiliations:** ^1^ Department of Gastrointestinal Medical Oncology, The University of Texas M.D. Anderson Cancer Center, Houston, TX, USA; ^2^ Department of Biostatistics, The University of Texas M.D. Anderson Cancer Center, Houston, TX, USA; ^3^ Department of Pathology, The University of Texas M.D. Anderson Cancer Center, Houston, TX, USA; ^4^ Department of Diagnostic Imaging, The University of Texas M.D. Anderson Cancer Center, Houston, TX, USA; ^5^ Department of Gastroenterology, The University of Texas M.D. Anderson Cancer Center, Houston, TX, USA; ^6^ Department of Radiation Oncology, The University of Texas M.D. Anderson Cancer Center, Houston, TX, USA; ^7^ Department of Surgical Oncology, The University of Texas M.D. Anderson Cancer Center, Houston, TX, USA; ^8^ Department of Medical Oncology, Princess Margaret Cancer Centre, University of Toronto, Toronto, ON, Canada

**Keywords:** localized gastric adenocarcinoma, cancer surveillance, cost-effectiveness analysis, imaging studies, esophagogastroduodenoscopy

## Abstract

**Purpose:**

After therapy of localized gastric adenocarcinoma (GAC) patients, the costs of surveillance, relapse patterns, and possibility of salvage are unknown.

**Materials and Methods:**

We identified 246 patients, who after having a negative peritoneal staging, received therapy (any therapy which included surgery) and were surveyed (every 3–6 months in the first 3 years, then yearly; ∼10 CTs and ∼7 endoscopies per patient). We used the 2016 Medicare dollars reimbursed as the “costs” for surveillance.

**Results:**

Common features were: Caucasians (57%), men (60%), poorly differentiated histology (76%), preoperative chemotherapy (74%), preoperative chemoradiation (59%), and had surgery (100%). At a median follow-up of 3.7 years (range, 0.1 to 18.3), the median overall survival (OS) was 9.2 years (95% CI, 6.0 to 11.2). Tumor grade (*p* = 0.02), p/yp stage (*p* < 0.001), % residual GAC (*p* = 0.05), the R status (*p* = 0.01), total gastrectomy (*p* = 0.001), and relapse type (*p* = 0.02) were associated with OS. Relapse occurred in 79 (32%) patients (only 8% were local-regional) and 90% occurred within 36 months of surgery. P/yp stage (*p* < 0.001) and total gastrectomy (*p* = 0.01) were independent prognosticators for OS in the multivariate analysis. Only 1 relapsed patient had successful salvage therapy. The estimated reimbursement for imaging studies and endoscopies was $1,761,221.91 (marked underestimation of actual costs).

**Conclusions:**

The median OS of localized GAC patients was excellent with infrequent local-regional relapses. Rigorous surveillance had a low yield and high “costs”. Our data suggest that less frequent surveillance intervals and limiting expensive investigations to symptomatic patients may be warranted.

## INTRODUCTION

Gastric adenocarcinoma (GAC) continues to be a significant health burden worldwide and is the third leading cause of cancer death [1]. Even in the localized setting, the outcomes of GAC patients generally remain poor. [2, 3] However, adjunctive approaches have increased the cure rate by approximately 10%. [2–5]. Therefore, the current approach to localized GAC (>cT1b) is to use a either preoperative or postoperative strategy to maximize the benefits of surgery [6]. There are regional preferences in the utilization of these approaches. After completion of such therapies, patients are surveyed by a variety of algorithms based on local practices/preferences. Since evidence-based surveillance is currently not possible because of the lack of effectiveness data, the National Comprehensive Cancer Network guidelines for surveillance are broad to accommodate various participating institutions’ routines (www.nccn.org) [6]. Surveillance is costly and anxiety generating. Accurate estimates of the costs are not available for GAC surveillance. In addition to the albeit small, inherent risks of testing itself (procedure complications and radiation exposure), there are emerging concerns about the excessive use of expensive investigations without documentation of benefit [7]. For GAC, the role of surveillance has not been formally studied and the endpoints are also not defined. We have arbitrarily defined “successful” salvage as those patients who upon having an “actionable” recurrence have salvage therapy (example, chemoradiation, surgery, or both) and survive at least 2 years. However, additional discussions are needed to develop acceptable and meaningful endpoints from surveillance.

The purpose of our study was to assess the outcomes of a routine, frequent, and costly surveillance strategy that was implemented at our institution. We also calculated the associated costs (by using the Medicare reimbursements) of these expensive surveillance tools (e.g., computerized tomography and endoscopy). To our knowledge, such results have not been described in the literature.

## RESULTS

### Patient characteristics

A total of 246 patients with GAC were identified with dates of surgery between May 1995 and August 2014. Table 1 shows that patients were primarily Caucasian (57%) men (60%), with a mass visualized on esophagogastroduodenoscopy (EGD) (78%), and poorly differentiated GAC (76%). Most patients (59%) received preoperative chemoradiation, while 11% had preoperative chemotherapy only. The remaining patients either had some form of adjuvant therapy (8%) or were appropriate for surgery only (22%).

**Table 1:** Patient characteristics

Characteristics	*N* (%)
All	
	246 (100%)
**At Diagnosis**	
Age at Diagnosis - median (min,max)	
*N* = 246	61.3 (26.2,87.4)
Gender	
Female	99 (40%)
Male	147 (60%)
Race	
White	139 (57%)
Black or African American	28 (11%)
Hispanic	47 (19%)
Asian	26 (11%)
Other	6 (2%)
Alcohol	
Frequent	50 (20%)
Past	29 (12%)
Rarely	75 (30%)
Never	92 (37%)
Smoking	
Nonsmoker	108 (44%)
Nonsmoker Quit	100 (41%)
Smoker	38 (15%)
Length of Tumor (cms)	
≤ 3	60 (24%)
>3	101 (41%)
Missing	85 (35%)
Location of Tumor	
Gastric	182 (74%)
AEG2	3 (1%)
AEG3	61 (25%)
Baseline Stage for AEG3 & Gastric	
I	53 (22%)
II	88 (36%)
III	94 (38%)
IV	9 (4%)
Missing	2 (1%)
Mass	
Yes	192 (78%)
No	50 (20%)
Missing	4 (2%)
Tumor Grade	
G1 Well-Differentiated	2 (1%)
G2 Moderately Differentiated	53 (22%)
G3 Poorly Differentiated	188 (76%)
GX Undetermined Grade	2 (1%)
Missing	1 (0%)
Tumor Histology	
Adenocarcinoma	245 (100%)
Carcinoma Undetermined	1 (0%)
Adenocarcinoma Subtype	
SRC- Signet ring cells	107 (44%)
M & SRC	18 (7%)
M- Mucinous	3 (1%)
NE- neuro endocrine	1 (0%)
NOS- Not Otherwise Specified	116 (47%)
p/yp Stage	
0	31 (13%)
I	65 (26%)
II	81 (33%)
III	58 (24%)
IV	7 (3%)
Missing	4 (2%)
% Residual cancer	
P0	31 (13%)
P1	70 (28%)
P2	40 (16%)
PX	101 (41%)
Missing	4 (2%)
Treatment	
Treatment	
Preoperative Chemotherapy	28 (11%)
Preoperative Chemoradiation	145 (59%)
Other	20 (8%)
None	53 (22%)
R(Margin)	
R0 Resection	212 (86%)
R1 Resection	29 (12%)
Missing	5 (2%)
Total Gastrectomy	
Yes	85 (35%)
No	160 (65%)
Missing	1 (0%)

AEG = Adenocarcinoma of Esophagogastric Junction; CTRT = Chemoradiation.

### Imaging and EGD at the time of relapse

Table 2 shows the relapse outcomes and how they were diagnosed (imaging and/or EGD) overall and by margin status at resection. A total of 79 patients relapsed, 17 (22%) of patients had R1 resection and and 60 (78%) of patients had R0 resection and 2 patients had unknown margin status. Of the 79 relapsed patients, 73 (92%) had DM and 6 (8%) had LRR only. For imaging, 65 patients had CTs, 9 had PET-CTs, and 3 had MRI. Imaging detected 70 of the 79 relapses (rest diagnosed by EGD). Of the relapsed patients, 51 (65%) had EGD at relapse and 7 (9%) patients had a positive biopsy. As expected, R1 patients were more likely to have an endoscopy done at relapse with histologically confirmed relapse.

**Table 2:** Relapse outcome for CT and endoscopy by R margin resection status

Characteristics	Resection Status		
	R0 *N* (%)	R1 *N* (%)	All Patients* *N* (%)
Relapse			
Yes	60 (28%)	17 (59%)	79 (32%)
No	149 (70%)	12 (41%)	162 (66%)
Lost to Follow-up	3 (1%)	0 (0%)	4 (2%)
Missing	0 (0%)	0 (0%)	1 (0%)
Relapse Location			
Distant	56 (93%)	16 (94%)	73 (92%)
Luminal/Regional	4 (7%)	1 (6%)	6 (8%)
Type of Imaging Study			
CT-Contrast	48 (80%)	15 (88%)	65 (82%)
MRI	3 (5%)	0 (0%)	3 (4%)
PET-CT	7 (12%)	2 (12%)	9 (11%)
Missing	2 (3%)	0 (0%)	2 (3%)
Failure Suspected by Imaging			
Yes	53 (88%)	16 (94%)	70 (89%)
No	5 (8%)	1 (6%)	7 (9%)
Missing	2 (3%)	0 (0%)	2 (3%)
Endoscopy Done at Relapse			
Yes	36 (60%)	13 (76%)	51 (65%)
No	23 (38%)	4 (24%)	27 (34%)
Missing	1 (2%)	0 (0%)	1 (1%)
Biology Confirmed			
Yes	3 (5%)	3 (18%)	7 (9%)
No	33 (55%)	10 (59%)	44 (56%)
Not Done	24 (40%)	4 (24%)	28 (35%)

*Five patients overall and 2 relapsed patients are missing R Margin status so rows will not always sum to the All Patients numbers

Supplementary Table 1 shows relapses among the R0 resected patients by EGD vs. imaging and for the 4 patients with luminal/regional relapse. As expected in the R0 patients, imaging detected more relapses (88%) than did EGD (5%). Among the 4 R0 patients with LRRs, 2 patients had abnormal imaging and EGD diagnosed 2. Supplementary Table 2 shows the R status for relapsed patients. There was no association between R margin and relapse location.

### Overall survival (OS)

The median follow-up time among survivors was 3.7 years (range, 0.1 to 18.3 years). Eighty-eight patients (36%) died and 158 (64%) were alive at last follow-up. The median OS was 9.2 years (95% CI: 6.0, 11.2). The 5-year OS rate was 64% (SE = 4%; Supplementary Figure 1). Supplementary Table 3 shows OS by patient characteristics. Tumor grade (*p* = 0.02), p/yp stage (*p* < 0.001), % residual GAC (*p* = 0.05), the R margin (*p* = 0.01), total gastrectomy (*p* = 0.001), and relapse location (*p* = 0.02) were associated with OS. Patients with well- or moderately- differentiated GACs had longer OS than to those with poorly differentiated GACs (86% vs. 58% alive at 5 years, respectively). Patients with yp stage 0 (pathologic complete response = pCR) or yp stage I lived longer than those with yp stage II, III, or IV (90%, 88%, vs, 57%, 38%, and 0% alive at 5 years, respectively). Similarly, patients with P0 GAC (pCR) lived longer than those with P1 GAC (1% to 50% residual GAC) or P2 GAC (> 50% residual GAC; 89%, 61%, and 53% alive at 5 years, respectively). Patients who had R0 resection lived longer than those with R1 resection (67% vs. 40% alive at 5 years, respectively). Patients with subtotal gastrectomy lived longer than those with total gastrectomy (73% vs. 50% alive at 5 years, respectively).

Table 3 shows the multivariate analysis results for OS. Age at diagnosis, location of GAC, baseline stage, tumor grade, p/yp stage, the R margin, and total gastrectomy were included in a multivariate model. p/yp stage and total gastrectomy remained significant in the reduced model. Patients with advanced p/yp stage (*p* < 0.001) and total gastrectomy (*p* = 0.01) were at higher risk of death.

**Table 3:** Multivariate Cox proportional hazards models for RFS, TTR and OS

		Full Model			Reduced Model		
Characteristic		HR	95% CI	*P*-value	HR	95% CI	*P*-value
Recurrence-Free Survival (RFS)			(E/N = 103 /235)			(E/N = 108 /242)	
Age at Dx		0.99	(0.97, 1.01)	0.25			
Location of Tumor	Gastric vs. AEG 2/3	0.78	(0.47, 1.30)	0.34			
Baseline Stage	II vs. I	1.21	(0.64, 2.26)	0.83			
	III/IV vs. I	1.10	(0.59, 2.02)				
Tumor Grade	G3 Poorly Differentiated vs. G1/G2 Well/Moderately Differentiated	1.74	(0.93, 3.26)	0.08			
p/yp Stage	I vs. 0	0.93	(0.34, 2.55)	< 0.001	0.79	(0.32, 1.94)	< 0.001
	II vs. 0	3.06	(1.27, 7.41)		3.02	(1.41, 6.48)	
	III vs. 0	5.04	(2.01, 12.66)		4.82	(2.24, 10.37)	
	IV vs. 0	31.79	(9.25, 109.27)		34.66	(11.93, 100.72)	
R Margin	R1 Resection vs. R0 Resection	1.29	(0.76, 2.18)	0.34			
Total Gastrectomy	Yes vs. No	1.24	(0.81, 1.89)	0.32			
**Time to Recurrence**			**(E/N=74 /235)**			**(E/N=77 /242)**	
Age at Dx		0.98	(0.96, 1.00)	0.02	0.98	(0.96, 0.99)	0.01
Location of Tumor	Gastric vs. AEG 2/3	0.87	(0.48, 1.59)	0.65			
Baseline Stage	II vs. I	1.43	(0.64, 3.23)	0.60			
	III/IV vs. I	1.48	(0.69, 3.17)				
Tumor Grade	G3 Poorly Differentiated vs. G1/G2 Well/Moderately Differentiated	2.26	(0.95, 5.41)	0.07			
p/yp Stage	I vs. 0	0.66	(0.11, 4.04)	<0.001	0.75	(0.13, 4.50)	< 0.001
	II vs. 0	6.00	(1.41, 25.53)		8.44	(2.02, 35.18)	
	III vs. 0	10.43	(2.39, 45.54)		14.47	(3.46, 60.55)	
	IV vs. 0	46.14	(8.30, 256.67)		81.31	(16.00, 413.27)	
R Margin	R1 Resection vs. R0 Resection	1.17	(0.64, 2.13)	0.60			
Total Gastrectomy	Yes vs. No	1.02	(0.62, 1.67)	0.95			
**Overall Survival**			**(E/N=84 /235)**			**(E/N = 86 /241)**	
Age at Dx		1.01	(0.99, 1.03)	0.50			
Location of Tumor	Gastric vs. AEG 2/3	0.84	(0.47, 1.48)	0.54			
Baseline Stage	II vs. I	1.01	(0.51, 1.98)	0.89			
	III/IV vs. I	0.89	(0.46, 1.74)				
Tumor Grade	G3 Poorly Differentiated vs. G1/G2 Well/Moderately Differentiated	1.81	(0.91, 3.61)	0.09			
p/yp Stage	I vs. 0	0.84	(0.30, 2.34)	< 0.001	0.78	(0.31, 1.94)	< 0.001
	II vs. 0	1.95	(0.79, 4.84)		1.84	(0.84, 4.06)	
	III vs. 0	3.63	(1.40, 9.40)		3.61	(1.65, 7.91)	
	IV vs. 0	22.91	(6.32, 83.04)		21.50	(6.74, 68.57)	
R Margin	R1 Resection vs. R0 Resection	1.29	(0.71, 2.34)	0.40			
Total Gastrectomy	Yes vs. No	1.66	(1.03, 2.67)	0.04	1.73	(1.11, 2.67)	0.01

E = Events; N = Total; HR = Hazard Ratio; CI = Confidence Interval.

### Relapse free survival (RFS)

**Figure 1:**
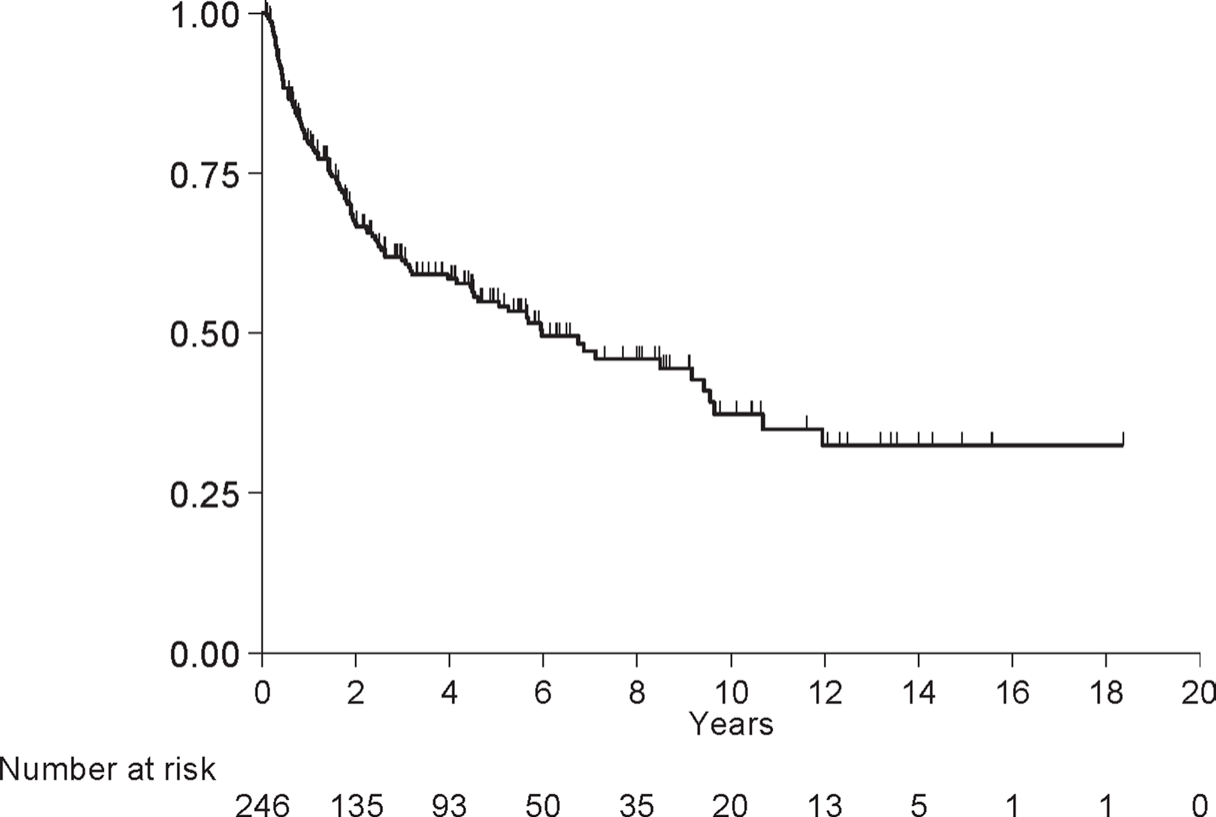
Relapse free survival

One hundred eleven (45%) patients experienced a relapse or died and 135 (55%) were relapse-free at last follow-up. The median RFS was 6.0 years (4.5, 9.4) and the 5-year RFS was 55% (SE = 4%; Figure 1). Supplementary Table 3 shows the RFS data by patient characteristics. RFS was associated with baseline stage (*p* = 0.03), tumor grade (*p* = 0.01), p/yp stage (*p* < 0.001), % residual GAC (*p* = 0.01), the R margin (*p* = 0.01), and total gastrectomy (*p* = 0.03). Eighty-one percent of patients with G1/G2 tumors were alive without relapse at 5 years compared to 48% of patients with G3 tumors. Patients with p/yp stage 0 or I were lived longer than patients with p/yp stage II, III or IV (90%, 86%, 43%, 29%, and 0% alive without relapse at 5 years, respectively). A total of 60% of patients who had R0 resection and 24% of patients who had R1 resection were alive without relapse at 5 years. Patients with subtotal gastrectomy were most likely to live without relapse than those with total gastrectomy (61% vs. 45% alive at 5 years, respectively). p/yp stage was an independent prognosticator in the reduced multivariate model (*p* < 0.001; Table 3).

### Time to relapse (TTR)

**Figure 2:**
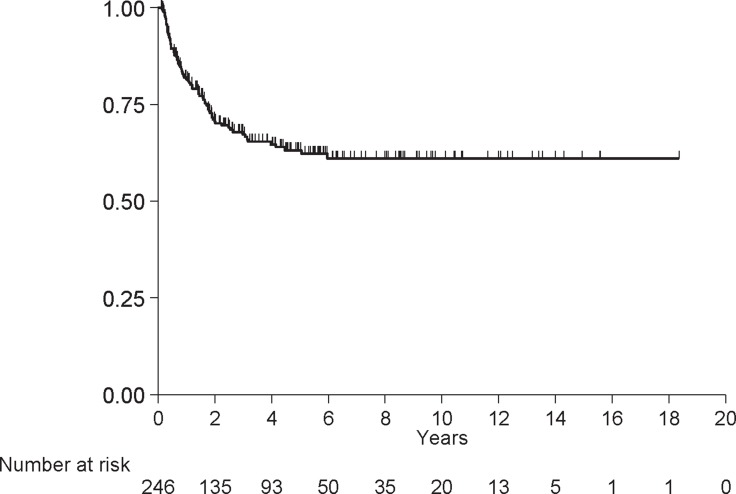
Time to relapse

Seventy-nine (32%) patients relapsed and 167 (68%) did not. The median TTR was not reached and the 5-year estimate was 63% (SE = 3%; Figure 2). Supplementary Table 3 presents the TTR results by patient characteristics. Age at diagnosis (*p* = 0.03), baseline stage (*p* = 0.01), tumor grade (*p* = 0.01), p/yp stage (*p* < 0.001), % residual GAC (*p* = 0.01), and R margin (*p* = 0.003) were associated with TTR. 85% of patients with G1\G2 tumors did not relapse at 5 years vs. 57% of patients with G3 tumors did relapse. Table 2 shows the multivariate analysis for age at diagnosis, location of GAC, baseline stage, tumor grade, p/yp stage, R margin and total gastrectomy (full model). Age at diagnosis and p/yp stage remained significant in the reduced model.

Supplementary Table 4 presents the counts of events and censors for deaths and relapse by time intervals. Most relapses occurred between 0 and 24 months (65/79).

### Surveillance CT scan and endoscopy

Table 4 shows a summary of surveillance CTs and EGDs for all patients and by R margin. Of the 246 patients had a total of 1053 CTs. A total of 147 (60%) patients had only negative results from CTs (730 scans), 43 (17%) patients ever had a positive result (4% of all CTs), and 47 (19%) patients had at least one suspicious result. Out of 56 suspicious CTs, 18 (32%) were subsequently negative, 31 (55%) were true positive, and 7 (13%) scans remained suspicious. Among R0 patients, 30/916 (3%) of CTs were positive while 12/126 (10%) were positive among R1 patients. Only 164 (67%) patients had at least one EGD with a total of 403 EGDs and of these only 3 were positive. Two patients’ first EGD was positive, and third patient had a negative EGD but the subsequent one was positive, therefore, 4 EGDs in 3 patients detected GAC relapse. Only 3/403 (1%) surveillance EGDs were positive, and 1/349 (0.3%) was positive in R0 patients and 2/48 (6%) positive in R1 patients. A total of 150 (61%) patients had a CT within 45 days of an EGD, leading to 321 matched tests. Of these patients, 31 (13%) had at least one discordant result.

**Table 4:** Summary of surveillance CT scan and endoscopy

		All Patients		R0 Patients		R1 Patients	
		Pts *N* (%)	Screens *N*	Pts *N* (%)	Screens *N*	Pts *N* (%)	Screens *N*
	All Patients	246		212		29	
CT Scans							
	All CT Scans*	228 (93%)	1053	196 (92%)	916	29 (100%)	126
	All Scans Negative	147 (60%)	730	132 (62%)	663	13 (45%)	57
	Any Negative (But Not All)	63 (26%)	224	51 (24%)	179	12 (41%)	45
	Any Suspicious**	47 (19%)	56	38 (18%)	44	9 (31%)	12
	Negative		18		17		1
	Positive		31		23		8
	Suspicious		7		4		3
	Any Positive	43 (17%)	43	30 (14%)	30	12 (41%)	12
Endoscopy							
	All Endoscopies*	164 (67%)	403	144 (68%)	349	19 (66%)	48
	All Endoscopy Negative	161 (65%)	399	143 (67%)	348	17 (59%)	45
	Ever Positive***	3 (1%)	4	1 (0%)	1	2 (7%)	3
Match****							
	All Match Screens	150 (61%)	321	131 (62%)	277	18 (62%)	39
	All Consistent Negative	117 (48%)	255	103 (49%)	218	13 (45%)	32
	All Consistent Ever Positive***	2 (1%)	3	0 (0%)	0	2 (7%)	3
	Mismatched	31 (13%)	63	28 (13%)	59	3 (10%)	4

*All CT scans and all endoscopies include those done within 5.5 years after resection date with the following exceptions: a) scans done more than 45 days after relapse, or b) any CT scan after a positive CT scan was excluded from this study.

**The resolution of the suspicious CT scan was defined by these rules in the following order:

Step 1: If this suspicious result was found within 60 days before or 45 days after relapse, it was defined as “Positive”.

Step 2: Define by using the next CT scan results.

Step 3: If specific keywords (such as metastasis, mets, chemo and positive) were found in the intervention or comment, then it was defined as “Positive”. Similarly, if specific keywords (such as negative) were found in the intervention or comment, then it was defined as “Negative”.

***This includes all endoscopies for patients with any positive endoscopy, so “additional screens” includes the negatives received prior to the positive.

****We matched each screening endoscopy with a CT scan done within 45 days. If there were multiple CTs available, then the nearest one was selected.

Supplementary Table 5 presents the 321 pairs of CT/EGD and the patient's true positive status. There were 8 (3%) false positives by CTs. Of the 27 true positives, 2 (7%) were diagnosed by EGD and CT, while 23 (85%) were by CT only. Two (7%) relapsed patients were diagnosed neither by EGD nor CT.

Supplementary Table 6 shows CTs and EGDs for R0 patients who relapsed. The 60 R0 relapsed patients had 188 CTs (30 CTs were positive, and 29 were suspicious). These 60 patients had 65 EGDs but only 1 was positive. Ten patients who relapsed were not diagnosed by EGDs or CTs. In a subgroup of 4 patients with LRR, 2 were diagnosed by CTs, 1 was confirmed after suspicious CT and 1 was not identified by a surveillance test. 15 CTs and 7 EGDs were performed in these 4 patients (3 had peritoneal carcinomatosis leading to aborted salvage surgery).

### Cost of surveillance

There were a total of 1053 CTs and 403 EGDs. Typical CT charges include: technical and professional charges for a thorax CT with dye (CPT code 71260) and for abdominal/pelvis CT with contrast (74177), and a clinic visit to review results (99213). The estimated reimbursement is $1,133.14 for each of these. A typical EGD charges include: technical and professional charges for EGD biopsy (43249) and pathology (88305), anesthesia (A740), and a clinic visit to review results. Thus the reimbursement for one EGD is $1,274.79. It should be emphasized that this amount does not include the charges of the preparation medications or any complications from biopsy or anesthesia. By performing CTs and EGDs (counting one clinic visit when both are done) the estimated 5-year Medicare reimbursement is $20,996.53 for a patient without relapse. This is an underestimate of the total charges. Using these the 2016 Medicare reimbursement rates, the total reimbursement for all imaging studies and EGDs was ∼$1,761,221.91. These are gross underrepresentation of actual costs and of the non-Medicare settings. These charges do not include 10 patients who were not surveyed at our institution. It is noteworthy that only one patient was successfully salvaged by our definition.

## DISCUSSION

The adjunctive therapies used for patients with localized GAC vary geographically. In North America and Europe, results from the INT-0116 [3] and British Medical Research Council Adjuvant Gastric Cancer Infusional Chemotherapy (MAGIC) trials have established the standard of care [2]. In Asia, adjuvant chemotherapy following a D2 resection is considered the standard [4, 8]. The strategy developed at our institution is currently being investigated in the prospective TOPGEAR and CRITICS-II trials [9, 10]. The 5-year OS rate was 64% (SE = 4%; Supplementary Figure 1) for our patients. The 5-year OS rate from large randomized trials was 35–45%, and although our study cannot be compared to these trials their results need to be considered [2, 11]. More interestingly in a similar analysis to ours with a similar patient population, Spolverato et al. [12] reported a 5-year survival rate of 39.3%. Their study was a multicenter experience (major centers in the US) with the majority of patients receiving adjuvant chemotherapy or radiation (only 2.9% had preoperative radiation). Similar to our study the majority of their patients had either clinical stage II or III GAC. With a median follow-up of 28.9 months, 29.9% of their patients developed a relapse with 75.8% had some component of DM and 24.2% had LRR.

Overall, 32% of our patients developed a relapse (92% with DM and only 8% with LRR) regional. Other investigators have reported three main patterns of relapse that include: LRR, peritoneal and DM [13, 14] but with a heterogeneous distribution [15–19]. LRRs are considerably reduced by surgery and remain a significant problem without surgery. Therefore we believe that the low LRR rate reported in our study is at least partially explained by the fact that high volume surgeons perform all gastric cancer surgeries.

In our study, RFS was associated with baseline stage (*p* = 0.03), tumor grade (*p* = 0.01), p/yp stage (*p* < 0.001), % residual GAC (*p* = 0.01), R margin (*p* = 0.01), and total gastrectomy (*p* = 0.03). This is in agreement with observations by several other investigators [13, 20–22]. Relatively few studies have examined timing of relapse in GAC [12, 14, 20]. Similar with our study, others have noted that the majority of relapses occur within 2–3 years.

The unique aspect of our study is the analysis of the value of surveillance and reimbursements associated with an aggressive follow-up schedule. The salvage strategy is not effective since only one patient could have it. The routine use of EGDs in our patients was also ineffective and we have abandoned EGD as a surveillance tool. The overall reimbursements, based on 2016 Medicare rates, of surveillance was $20,996.53/patient for a total of $1,761,221.91 for all patients. These excluded 10 patients and costs also do not reflect charges at non-Medicare rates, which are commonly much higher.

Our study has limitations. First, it is a retrospective analysis with its associated potential bias and second it a single center experience and not generalizable. However, our study does have a number of strengths. These include: (1) demonstration that salvage strategy is not effective, (2) the benefit of EGD as surveillance is limited or non-existent, and (3) the reimbursements for surveillance are modest but the actual costs are likely prohibitive. Our patients’ outcomes were excellent, however, the preoperative chemoradiation strategy does not have level-1 evidence and has been labeled category 2B in the current NCCN GAC guidelines [6].

In conclusion, our data from a large cohort of patients with GAC show that LRRs are infrequent (8%). Successful salvage is rare and therefore routine surveillance, especially EGD, is not cost-effective. Further prospective research is needed to provide objective data for the cost effectiveness for surveillance.

## MATERIALS AND METHODS

### Patient selection

From our prospectively maintained database on GAC in the Department of Gastrointestinal Medical Oncology at The University of Texas MD Anderson Cancer Center, we identified consecutive patients who, between 1995 and 2014, had histologically confirmed GAC and successfully completed local/adjunctive therapy. The majority of patients received preoperative chemotherapy or preoperative chemotherapy/chemoradiation. All patients had baseline and pre-surgery staging (for patients receiving preoperative therapy) that included imaging studies, esophagogastroduodenoscopy (EGD) with biopsies and endoscopic ultrasonography. Imaging studies included chest and abdomen computed tomography (CT) and/or positron emission tomography (PET) with CT. All patients had a negative baseline laparoscopic peritoneal staging. Before proceeding with therapy, each patient was seen by experts from appropriate disciplines and then discussed by the multidisciplinary team (consisting of radiologists, gastroenterologists, surgical oncologists, radiation oncologists, pathologists, nutritionists, geneticists [when appropriate], and medical oncologists). Clinical staging was based on American Joint Committee Classification (AJCC), 6th edition [23] and pathologic staging was based on AJCC, 7th edition [24]. The institutional review board approved this analysis.

### Therapy

The preferred strategy at our institution includes 2 months of chemotherapy (a fluoropyrimidine and platinum compound) followed by chemoradiation (fluoropyrimidine plus 45 Gy in 25 fractions) [25]. Alternative therapies included preoperative chemotherapy or adjuvant chemoradiation [3]. Patients who developed only a local-regional relapse (LRR) were re-discussed in the multidisciplinary conference to develop a consensus salvage strategy.

### Surveillance after therapy

Each patient was generally surveyed as follows: patient visits were performed every 3 months for the first year, then every 6 months for 2 additional years, and then once a year for at least 5 years. An imaging study (CT or PET-CT), and blood tests were performed at each visit. EGD and biopsies were performed every 6 months in the first 24 months and then once a year.

First and second relapses, distant metastases (DM, with or without LRR) or only LRR (actionable) were recorded. The surveillance methods or diagnostic methods (triggered by surveillance or new symptom) were tabulated. The survival information was obtained from our tumor registry, medical records, and/or the Social Security database.

GAC patients treated between May 1995 and April 2014 were reviewed for dates of surveillance CT scans and EGDs occurring between surgery and 5.5 years post-surgery to account for variability in visits after the planned 5-year window. Only surveillance tests performed at our institution were included in the cost-analysis.

### Statistical methods

Patient characteristics and relapse outcomes were summarized using descriptive statistics. Overall survival (OS) was defined as the number of years between surgery and death from any cause, and was censored at last follow-up for living patients. Relapse free survival (RFS) was defined as number of years between the date of surgery and relapse or death. Patients who were alive without relapse were censored at the date of last follow-up. Time to relapse (TTR) was defined as the number of years between date of surgery and relapse, and was censored at last follow-up for patients without relapse. Survival curves were estimated using the Kaplan-Meier method [26] and median time was reported with a 95% confidence interval (CI). Univariate and multivariate Cox proportional hazards regression models [27] were used to assess the association between patient characteristics and OS, RFS or TTR. For relapse location, OS was only estimated among relapsed patients and was calculated from relapse date instead of surgery date. Age at diagnosis, location of GAC, baseline stage, tumor grade, p/yp stage, the R margin and total gastrectomy were included in a multivariate model (full model). Then, backward elimination was implemented until all remaining predictors had a *p*-value less than 0.05 (reduced model). Details of how surveillance CT scans and EGDs as well as Medicare cost estimation [28, 29] are detailed in the supplementary materials. Statistical analyses were performed in SAS 9.3 [The SAS Institute, Cary, NC, USA], and figures were created in Stata 13.1 [Stata Corp, College Station, TX, USA].

## SUPPLEMENTARY FIGURE AND TABLES




