# Supplementary material for: Patterns of relapse in patients with localized gastric adenocarcinoma who had surgery with or without adjunctive therapy: costs and effectiveness of surveillance
Source: Oncotarget. 2017 Jul 13;8(46):81430–40. doi: 10.18632/oncotarget.19226 (PMC5655297; doi:10.18632/oncotarget.19226)
Supplement: Supplementary file 1 [file oncotarget-08-81430-s001.pdf]

Patterns of relapse in patients with localized gastric adenocarcinoma who had surgery with or without adjunctive therapy: costs and effectiveness of surveillance

SUPPLEMENTARY MATERIALS

Identification of medicare cost components

The dates of each surveillance CT and endoscopy were sent for billing details +/− 5 days from each test date, collecting all technical and professional billing codes and descriptions in the time frame for each assessment. Then (EE and RS) reviewed the data to identify selection criteria from the descriptions. We identified those charges standardly related to CT or endoscopy and created a data set of CT and endoscopy professional and technical charges. For quality control in the process, an additional dataset of deleted items was also created and reviewed to confirm that deleted items were unrelated to standard surveillance practices, and everything kept was related. Once the related items were finalized, the Medicare cost amounts for the associated CPT codes of technical and professional charges were obtained. In order to choose which CPT code and cost to use, we looked at the frequency of each overlapping code, selected the one billed the most often, and chose the Houston, TX January 2016 Medicare code and dollar amount for that portion of the estimated surveillance cost. Finally, an estimated cost of each CT and each endoscopy using today's Medicare cost was calculated by summing the most frequent billing codes for each aspect of the testing procedures.

Overall screening cost estimation

The current surveillance recommendations for CT are scans and physician visits every 3 months in year 1 (4 scans), every 6 months in years 2 and 3 (4 scans) and then annually in years 4 and 5 (2 scans) for a total of 10 CT scans and physician visits. For endoscopy, the recommendations are every 6 months in the first 2 years (4 scans) and then annually for years 3-5 (3 scans) for a total of 7 endoscopies and clinic visits. To find the estimated 5-year cost for following these recommendations, we summed 10 CT scans, 7 endoscopies, and a clinic visit for each time point. When CT scans and endoscopies are recommended at the same time, we assumed one clinic visit could be used to review both results at once. Therefore we summed the cost of 10 CT scans, 7 endoscopies, and 10 clinic visits to estimate the cost of surveilling one patient for the full 5 years, assuming no relapse before the 5 year visit.

Determination of surveillance scans, results and matching

All Surveillance CT scans and endoscopies were identified in this study as those done within 5.5 years after surgery date with the following exceptions: a) scans done more than 45 days after relapse, or b) any CT scan after a positive CT scan was excluded from this study. The resolved result of a suspicious scan was defined by the following rules: First, if this suspicious result was found within 60 days before or 45 days after relapse, it was defined as "positive"; if still unresolved it was defined by using the next screen results; and finally if neither of these applied, the specific keywords (such as metastasis, mets, chemo and positive) were found in the intervention or comment, then it was defined as "positive". Similarly, if specific keywords (such as negative) were found in the intervention or comment, then it was defined as "negative". We matched each endoscopy with a CT scan done within 45 days. If there were multiple CTs available, then we chose the nearest one. To establish the accuracy of screens, we established the "true" screen results of the matched screens by the results of CT scans and endoscopies or presence of documented progression. If the result of either the CT scan or endoscopy was "positive", then the true status was determined as positive, or if a patient relapsed within 60 days after this pair of tests, the true status was "Positive". If both CT scans and endoscopies were "negative" and the patient did not relapse within 60 days, then the true result was determined as negative. If the CT scan was suspicious and the endoscopy was negative, then the resolved value of the suspicious scan was used to find the true result as described.

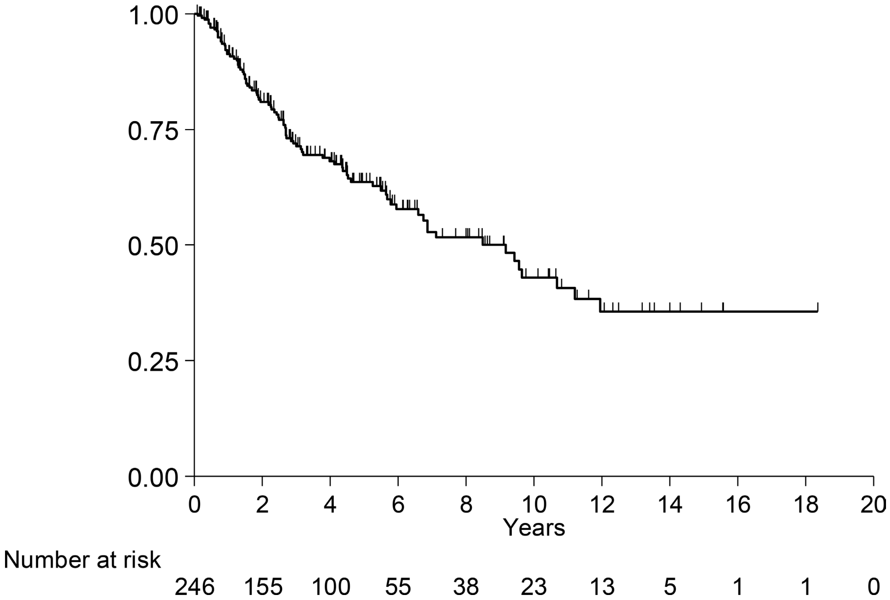

Supplementary Figure 1: Overall survival.

Supplementary Table 1: Identification of relapse by EGD versus imaging among R0 patients

	All Relapse				Luminal/Regional Relapse		
	Failure Suspected by Imaging				Failure Suspected by Imaging		
**Biology Confirmed by EGD**	**Yes** *N* **(%)**	**No** *N* **(%)**	**Missing**	**Total**	**Yes** *N* **(%)**	**No** *N* **(%)**	**Total**
Yes	1 (2%)	2 (40%)	0	3	0 (0%)	2 (100%)	2
No	31 (58%)	2 (40%)	0	33	2 (100%)	0 (0%)	2
Not Done	21 (40%)	1 (20%)	2 (100%)	24	0 (0%)	0 (0%)	0
Total	53	5	2	60	2	2	4

Supplementary Table 2: Margin status by relapse location

	R Margin			
**Relapse Location**	**R0 Resection** *N* **(%)**	**R1 Resection** *N* **(%)**	**Missing** *N* **(%)**	**Total**
Distant	56 (93%)	16 (94%)	1 (50%)	73
Luminal/Regional	4 (7%)	1 (6%)	1 (50%)	6
Total	60	17	2	79

Supplementary Table 3: Survival and recurrence outcomes by patient characteristics. See Supplementary_Table_3

Supplementary Table 4: Counts of non-relapsed patients by last follow-up time and relapsed patients by relapse time

Time	Interval		Total
	Last Follow-Up No Relapse	Relapse	Relapse
0–6 months	12	25	25
6–12 months	15	18	43
12–24 months	19	22	65
24–36 months	20	6	71
36–48 months	12	4	75
48–60 months	20	2	77
≥ 60 months	69	2	79
Ever	167	79	79

Supplementary Table 5: Screen results among matched screens

	CT Scans		Endoscopy		
Truth*	Positive/Suspicious *N* (%)	Negative *N* (%)	Positive *N* (%)	Negative *N* (%)	Total
Positive	25 (93%)	2 (7%)	2 (7%)	25 (93%)	27
Negative	8 (3%)	286 (97%)	0	294 (100%)	294
Total	33 (10%)	288 (90%)	2 (1%)	319 (99%)	321

*Truth is positive if disease is found by CT or endoscopy or patient relapsed within 60 days of negative scans. It is negative if endoscopy is negative and CT is negative or suspicious that does not resolve to positive and no relapse was identified within 60 days.

Supplementary Table 6: Numbers of CT and Endoscopy Surveillance Results among Relapsed R0 Patients

	Relapsed Patients	CT Scans			Endoscopy		All Screens
	N	Positive *N* (%)	Suspicious *N* (%)	Negative *N* (%)	Positive *N* (%)	Negative *N* (%)	Total
All Relapses	60	30 (16%)	29 (15%)	129 (69%)	1 (2%)	64 (98%)	253
Luminal/Regional Relapses	4	2 (13%)	1 (7%)	12 (80%)	0 (0%)	7 (100%)	22

Among the 60 relapsed R0 patients, 30 (50%) had positive CT scans, 1 (2%) had positive endoscopy, 19(32%) had no positive CT scan but at least one suspicious CT scan which resolved to positive by confirmation. and 10 (17%) did not have any positive or suspicious screening tests (including 1 never had any screening test). Among the 4 patients with luminal/regional relapse, 3 were identified by surveillance CTs, 2 positive and 1 suspicious that was confirmed to be positive. The other patient was identified separately from surveillance CT or endoscopy.

Supplementary Table 7: Relapse outcome by resection year

	Resection Year			
	Prior to 2007	2007–2010	2011–2014	All
**Characteristics**	***N* (%)**	***N* (%)**	***N* (%)**	***N* (%)**
Relapse				
Yes	30 (36%)	22 (29%)	27 (32%)	79 (32%)
No	51 (61%)	54 (70%)	57 (67%)	162 (66%)
Lost to Follow-up	2 (2%)	1 (1%)	1 (1%)	4 (2%)
Missing	1 (1%)	0 (0%)	0 (0%)	1 (0%)
Relapse Location				
Distant	27 (90%)	21 (95%)	25 (93%)	73 (92%)
Luminal/Regional	3 (10%)	1 (5%)	2 (7%)	6 (8%)
Type of Imaging Study				
CT-Contrast	25 (83%)	20 (91%)	20 (74%)	65 (82%)
MRI	1 (3%)	1 (5%)	1 (4%)	3 (4%)
PET-CT	2 (7%)	1 (5%)	6 (22%)	9 (11%)
Missing	2 (7%)	0 (0%)	0 (0%)	2 (3%)
Failure Suspected by Imaging				
Yes	24 (80%)	20 (91%)	26 (96%)	70 (89%)
No	4 (13%)	2 (9%)	1 (4%)	7 (9%)
Missing	2 (7%)	0 (0%)	0 (0%)	2 (3%)
Endoscopy Done at Relapse				
Yes	20 (67%)	14 (64%)	17 (63%)	51 (65%)
No	9 (30%)	8 (36%)	10 (37%)	27 (34%)
Missing	1 (3%)	0 (0%)	0 (0%)	1 (1%)
Biology Confirmed				
Yes	4 (13%)	2 (9%)	1 (4%)	7 (9%)
No	16 (53%)	12 (55%)	16 (59%)	44 (56%)
Not Done	10 (33%)	8 (36%)	10 (37%)	28 (35%)
R Margin Status at Resection prior to Relapse				
R0	24 (80%)	16 (73%)	20 (74%)	60 (76%)
R1	5 (17%)	6 (27%)	6 (22%)	44 (22%)
Missing	1 (3%)	0 (0%)	1 (4%)	2 (3%)
